# Personalia

**Published:** 1914-09-26

**Authors:** 


### Personalia.

Dr. J. M. Johnston, assistant school medical officer for Hornsey, having been called up for service with the R.A.M.C., the Education Committee have decided to appoint, during Dr. Johnston's absence, a temporary part-time officer to serve on five half-days a week.

The Epping Rural Council have recently received and accepted with much regret the resignation of Dr. Trevor Fowler, who has held the post of medical officer for the Epping district for forty-one years, and during that time it is said of him that he has received no word of criticism—surely a unique experience.

